# Supplementary material for: Facile Synthesis of Ultrastable Fluorescent Copper Nanoclusters and Their Cellular Imaging Application
Source: Nanomaterials (Basel). 2020 Aug 26;10(9):1678. doi: 10.3390/nano10091678 (PMC7558839; doi:10.3390/nano10091678)
Supplement: Supplementary file 1 [file nanomaterials-10-01678-s001.pdf]

nanomaterials

Supplementary Materials

Facile synthesis of ultrastable fluorescent copper nanoclusters and their cellular imaging application

Wei Yan [1], Jianqiao Zhang [2], Muhammad Abbas [3], Yulian Li [2], Syed Zajif Hussain [3], Shazia Mumtaz [3], Zhengwei Song [1], Irshad Hussain [3,*] and Bien Tan [2,*]

1. Ministry-of-Education Key Laboratory for the Green Preparation and Application of Functional Materials, Hubei Collaborative Innovation Center for Advanced Organic Chemical Materials, Hubei Key Laboratory of Polymer Materials, Faculty of Materials Science and Engineering, Hubei University, WuHan,430062,China; willieyancn2003@foxmail.com (W.Y.); songkukus@163.com(Z.S.);
2. Key laboratory of Material Chemistry for Energy Conversion and Storage, Ministry of Education, School of Chemistry and Chemical Engineering, Huazhong University of Science and Technology, Wuhan, 430074,China, jqzhang1991@163.com(J.Z.); 940639118@qq.com(Y.L.);
3. Department of Chemistry & Chemical Engineering, SBA School of Science & Engineering, Lahore University of Management Sciences(LUMS), Lahore, Pakistan, Abbas@UTDallas.edu(M.A.); syed.hussain@lums.edu.pk(S.Z.H); shaziamumtaz5@gmail.com(S.M.); Muhammad Abbas is in Department of Chemistry and Biochemistry, University of Texas now.

*Correspondence: bien.tan@mail.hust.edu.cn (B.T.); ihussain@lums.edu.pk (I.H.); Tel.: + 86-27-8755-8172 (B.T.); Fax: (0)86-(0)27-87544532 (B.T.)

Received: 1 August 2020; Accepted: 25 August 2020; Published: date

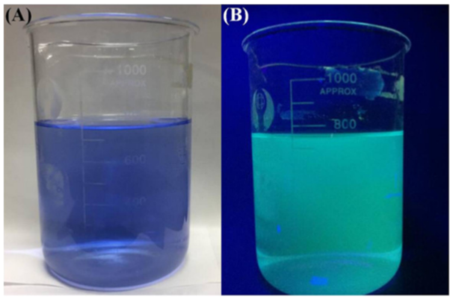

Figure 1. Photographs of Cu NCs under **(A)** ordinary daylight and **(B)** UV-irradiation.

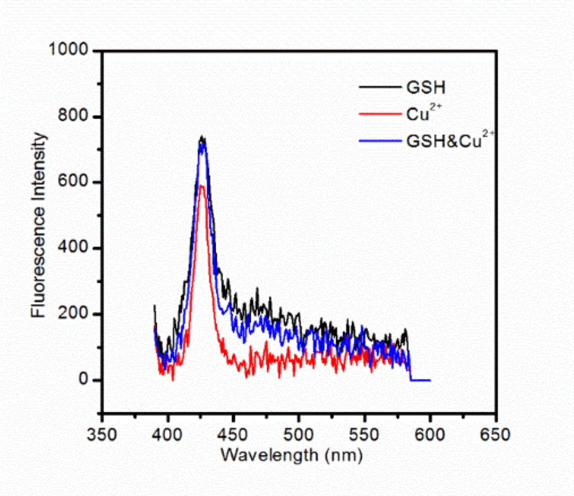

Figure 2. Fluorescence emission spectra of GSH, Cu^{2+} solution and mixture of GSH and Cu^{2+}.

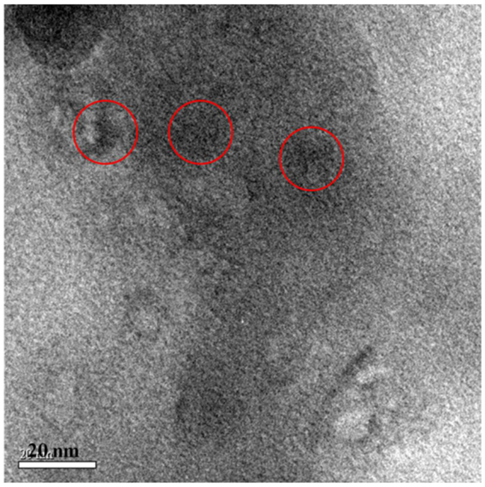

Figure 3. A high resolution TEM image of Cu NCs@GSH.

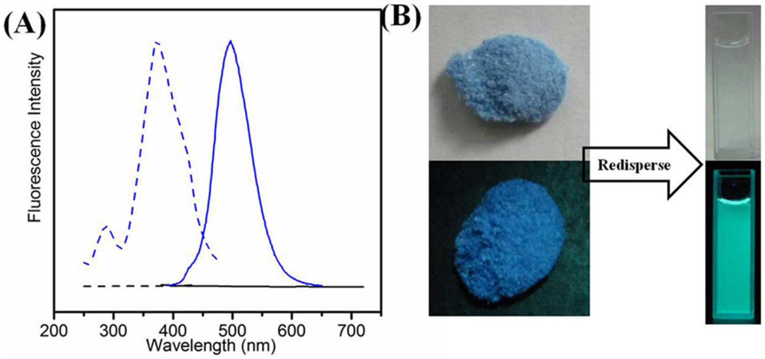

Figure 4. (A) Fluorescence excitation (dash line) and emission (solid line) spectra of Cu NCs@GSH in water (blue) and solid (black) state; **(B)** photographs of Cu NCs@GSH solid and redispersed in water under daylight (above) and 365 nm UV light (below).

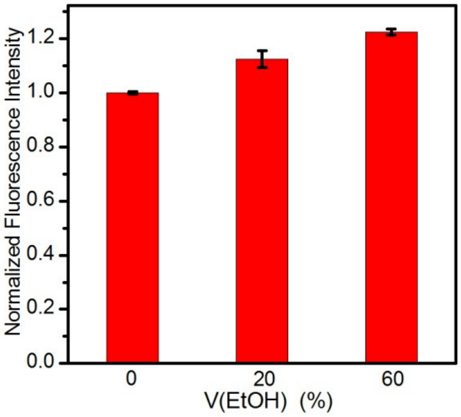

Figure 5. (A) Fluorescence emission spectra and **(B)** histogram of Cu NCs@GSH in different vol% ethanol: water mixture.

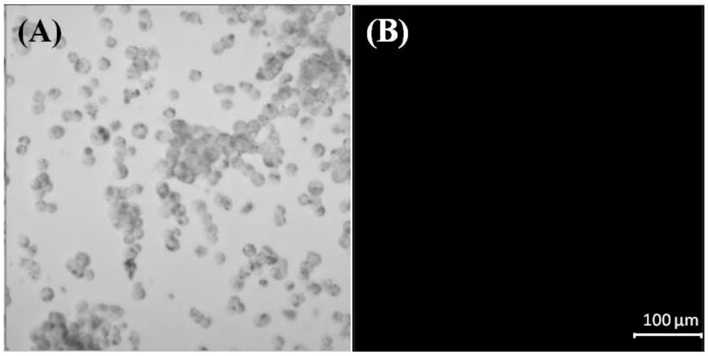

Figure 6. Images of HEK293 T cells incubated without Cu NCs obtained **(A)** under bright field and **(B)** at the excitation wavelength of 380 nm.

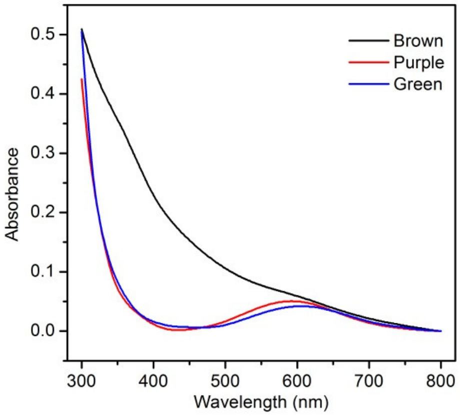

Figure 7. Absorption spectra of different color of Cu NCs solution.
